# 

**Published:** 1883-11

**Authors:** 


### HALL'S
### JOURNAL OF HEALTH
AND
### MISCELLANY.

MONTHLY.

E. H. GIBBS, A. M., M. D., EDITOR.

FOUNDED IN 1854.

### SUBSCRIPTIONS AND PREMIUMS.

THE subscription price of HALL'S JOURNAL OF HEALTH is $1 a year. News agents and booksellers will be allowed a commission of 25 cents for each subscription sent us. Any postmaster who sends us the name of one yearly subscriber, with $1, will himself receive the JOURNAL one year FREE. For each additional subscriber he will be entitled to the usual commission of 25 cents.

### To Publishers.

We will "club" HALL'S JOURNAL OF HEALTH with any other regular publication at 50 cents a year. We will not dictate as to the price of any other publication. Publishers can maintain their regular subscription price, and offer the JOURNAL OF HEALTH as a premium, if they desire to do so.

### Premiums.

In looking for a premium for subscribers we have unhesitatingly selected the NATIONAL STANDARD DICTIONARY as a most desirable book. A reliable dictionary is indispensable to every one. The wonder is how a dictionary containing 40,000 words—and in addition to this 700 illustrations, a Biograhical Register, foreign phrases geographical names, weights and measures of various nations, Mythological Dictionary and much other valuble information—can be handsomely bound and sold for $1, at which price we will send the Dictionary, post-paid, to any address in the United States or Canada Or we will send the Dictionary and also HALL'S JOURNAL OF HEALTH one year for $1.50.

That popular book of family medicine, DR. HALL'S HEALTH AT HOME, bound in cloth, we will send by mail, post-paid, on receipt of the regular price—$4.00—and we will in addition send HALL'S JOURNAL OF HEALTH, *free*, for one year to the purchaser, or to any other address desired. Or we will send the NATIONAL STANDARD DICTIONARY, DR. HALL'S HEALTH AT HOME, bound in cloth, and HALL'S JOURNAL OF HEALTH one year—all for $4.50. If leather binding is required for HEALTH AT HOME, 40 cents extra must be sent us. Remit by postal notes. E. H. GIBBS & Co.,

Publishers of HALL'S JOURNAL OF HEALTH,

New York City.

